# *Clostridioides* (*Clostridium*) *difficile* in neonatal foals and mares at a referral hospital

**DOI:** 10.1111/jvim.16094

**Published:** 2021-03-03

**Authors:** Jeffrey Scott Weese, Nathan Slovis, Joyce Rousseau

**Affiliations:** ^1^ Department of Pathobiology Ontario Veterinary College, University of Guelph Guelph Ontario Canada; ^2^ McGee Medical Center Hagyard Equine Medical Institute Lexington Kentucky USA

**Keywords:** gastrointestinal, infection control, infectious diseases, nosocomial

## Abstract

**Background:**

Understanding the epidemiology of *Clostridium difficile* is important for the development and assessment of infection prevention and control practices, as well as surveillance methods and interpretation of diagnostic testing results.

**Objective:**

Our objective was to longitudinally evaluate *C. difficile* shedding in neonatal foals and mares admitted to a referral hospital neonatal intensive care unit.

**Animals:**

Foals admitted to a neonatal intensive care unit, along with their dams.

**Methods:**

Rectal swabs were collected from mares and foals at admission, and then approximately every 3 days, when possible. Selective culture for *C. difficile* was performed and isolates were characterized by toxin gene PCR and ribotyping.

**Results:**

*Clostridium difficile* was isolated from 103/409 (25%) samples; 65/208 (31%) from foals and 38/201 (19%) from mares. Cumulatively, *C. difficile* was isolated from at least 1 sample from 50/113 (44%) foals and 30/97 (31%) mares. No association was found between hospitalization day and isolation of *C. difficile* (*P* = .13). Twenty‐three different ribotypes were identified, with ribotype 078 predominating. Fifteen foals had 2 positive samples during hospitalization. In only 6/15 (40%) foals was the same strain identified both times (5 ribotype 078 and 1 ribotype 012).

**Conclusions and Clinical Importance:**

*Clostridium difficile* is an important pathogen in adult horses and foals, and our findings highlight the complexity surrounding the epidemiology of this opportunistic pathogen. It can be found commonly, transiently, and cluster within a facility in the absence of identifiable disease occurrences or clusters.

**Abbreviations**
CA: community‐associated
CDI: *Clostridium difficile* infection
CDMN: *C. difficile* moxalactam norfloxacin
HA: hospital‐associated

## 1 INTRODUCTION

*Clostridioides* (*Clostridium*) *difficile* is a spore‐forming bacterium that is an important cause of colitis in adult horses and foals.^1^, ^2^, ^3^ In contrast to human medicine, where *C. difficile* infection (CDI) is diagnosed predominantly in older individuals in healthcare facilities, *C. difficile* and CDI are commonly found in horses outside of veterinary facilities.^4^, ^5^, ^6^, ^7^, ^8^, ^9^ However, the transmission dynamics of *C. difficile* in horses are poorly understood, both in veterinary facilities and on farms. Hospital‐associated (HA) CDI has been described in horses,^10^ but given the presence of *C. difficile* in horses without hospital exposure, studies that have not tested horses at admission cannot differentiate acquisition of *C. difficile* during hospitalization from development of disease from *C. difficile* that was present in the horse's gastrointestinal tract at admission. Variable (0%‐25%) rates of *C. difficile* shedding have been identified in adult horses without gastrointestinal disease on farms, with most studies reporting shedding rates of <5%.^1^, ^4^, ^5^, ^6^, ^7^, ^8^, ^9^, ^11^, ^12^ Reported shedding rates in healthy foals have been even more variable, but generally higher than reported for adults (2.6%‐44%).^1^, ^11^, ^13^

Understanding the epidemiology of *C. difficile* in hospitals is important for development and assessment of infection prevention and control practices, as well as surveillance methods and interpretation of diagnostic test results. Our objective was to longitudinally evaluate *C. difficile* shedding in neonatal foals and mares admitted to a referral hospital neonatal intensive care unit.

## 2 MATERIALS AND METHODS

### 2.1 Study population

Our study was performed at a referral hospital in Kentucky, USA. Foals that were admitted to the neonatal intensive care unit between February 22 and May 24, 2017, with or without accompanying mares, were eligible for inclusion. The neonatal intensive care unit predominantly manages foals ≤7 days of age, but older recumbent foals (eg, foals with botulism or head trauma) also are admitted. Foals with suspected infectious diarrhea or any other suspected infectious disease were excluded because they were admitted to a separate isolation unit. Rectal swabs (BBL CultureSwab Collection and Transport System, Becton Dickinson and Company, Sparks, Maryland) were collected from mares and foals at the time of admission, and approximately every 3 days during hospitalization. Clinical and antimicrobial use data were not reported.

### 2.2 Laboratory methods

The swabs were stored at −20°C before shipping overnight on ice to the laboratory. The entire swab was inoculated into 9 mL of *C. difficile* moxalactam norfloxacin (CDMN) enrichment broth (Oxoid Ltd; Nepean, ON, Canada) containing 0.1% sodium taurocholate. Samples were incubated anaerobically at 37°C for 7 days. After a 60‐minute alcohol shock, broth was subjected to centrifugation (3800*g* for 10 minutes) and the pellet was inoculated onto CDMN agar (Oxoid Ltd; Nepean, ON, Canada). After anaerobic incubation at 37°C for 48‐96 hours, colonies with morphology consistent with *C. difficile* were subcultured onto Columbia blood agar and identified based on characteristic morphology and odor of the colonies, Gram stain, and presence of L‐proline aminopeptidase activity (Remel Inc, Lenexa, Kansas).^14^ One colony per sample was collected for further study. Isolates were characterized by PCR ribotyping and PCR targeting genes encoding toxin A (*tcdA*), toxin B (*tcdB*) and binary toxin (*cdtA*, *cdtB*).^15^, ^16^ Isolates were compared to reference strains from the Cardiff‐European Centre for Disease Control Collection as well as an internal library, with further characterization by capillary ribotyping,^17^ with using the Webribo server (https://webribo.ages.at/).

### 2.3 Data analysis

Isolates were classified as community‐associated (CA) if detected from samples collected at the time of admission and HA if first detected ≥48 hours after hospitalization, after an initial negative sample.^18^ Source was not ascribed for positive samples collected after 48 hours of admission when an admission sample was not collected. Because of variation in sampling dates caused by logistical issues, the day of sampling was categorized as follows: admission, hospitalization 1 (H1; days 2‐4), hospitalization 2 (H2; days 5‐7), and hospitalization 3 (H3; days 8‐10).

Chi‐square or Fisher's exact tests were used for categorical comparisons. A *P <* .05 was considered significant.

## 3 RESULTS

### 3.1 Study population

Ninety‐seven mare and foal pairs, and an additional 16 unaccompanied foals were enrolled, corresponding to 210 horses (113 foals and 97 mares). All were thoroughbreds, apart from 2 mare‐foal standardbred pairs. Horses were from 76 farms, with a median of 2 horses (typically a single mare‐foal pair) admitted per farm (range, 1‐8). The median age of foals at admission was 24 hours (range, newborn‐30 days; interquartile range [IQR], 1 day). Eighty‐seven percent (98/113) of foals were ≤1 day of age and only 3 were >1 week of age. Mares ranged from 2 to 21 years of age (median, 9 years; IQR, 7 years). The ages of 2 mares were unknown.

Overall, 409 samples were collected; 208 from foals and 201 from mares. A median of 2 samples was collected from foals (range, 1‐5) and 3 from mares (range, 1‐4). One sample was collected from 39 (34%) foals and 30 (31%) mares, 2 from 58 (51%) foals and 42 (43%) mares, 3 from 13 (11%) foals and 21 (21%) mares, 4 from 1 (0.9%) foal and 4 (4.1%) mares, and 5 from 2 foals. Clinical status and clinic workload resulted in some variability in resampling timing.

### 3.2 *C. difficile* isolation

Overall, *C. difficile* was isolated from 103/409 (25%; 95% confidence interval [CI], 21%‐30%) samples, 65/208 (31%; 95% CI, 25%‐38%) from foals and 38/201 (19%; 95% CI, 14%‐25%) samples from mares. Cumulatively, *C. difficile* was isolated from at least 1 sample from 50/113 (44%; 95% CI, 35%‐53%) foals and 30/97 (31%; 95% CI, 23%‐41%) mares. The median number of positive samples from both foals and mares that were positive on at least 1 occasion was 1 (range, 1‐2).

### 3.3 *C. difficile* typing

Twenty‐three different ribotypes were identified, with ribotype 078 predominating. Overall, 102/103 (99%) of isolates were toxigenic, with 63 (62%) possessing *tcdA*, *tcdB*, and *cdtA/B*, and 39 (39%) possessing only *tcdA* and *tcdB*. Ribotypes detected more than once are presented in Table 1. Eight of the 14 (57%) ribotypes found only once possessed only *tcdA* and *tcdB*, whereas 5 (36%) possessed *tcdA*, *tcdB*, and *cdtA/B* and 1 was nontoxigenic. Ten of the 14 (71%) were found at admission whereas 4 (29%) were found during subsequent sampling.

**TABLE 1:** Ribotypes and isolate origin of *C. difficile* isolates found more than once (n = 89) from mares and foals at an equine hospital

Ribotype	Toxin gene	n (%)	Community‐associated	Hospital‐associated
078	*tcdA, tcdB, cdtA/B*	58 (56%)	36 (62%)	22 (37%)
056	*tcdA, tcdB*	7 (6.7%)	5 (71%)	2 (29%)
020	*tcdA, tcdB*	6 (5.9%)	2 (33%)	4 (67%)
MA7	*tcdA, tcdB*	5 (4.9%)	1 (20%)	4 (80%)
012	*tcdA, tcdB*	4 (3.9%)	3 (75%)	1 (25%)
AI‐37	*tcdA, tcdB*	3 (2.9%)	1 (33%)	2 (67%)
412	*tcdA, tcdB*	2 (2.0%)	2 (100%)	0
OVC‐O	*tcdA, tcdB*	2 (2.0%)	2 (100%)	0
AI‐82/1	*tcdA, tcdB*	2 (2%)	1 (50%)	1 (50%)

### 3.4 Epidemiological investigation

Fifteen of 96 (15.6%; 95% CI, 9.7%‐24%) mares and 31/113 (27%; 95% CI, 20%‐36%) foals had CA *C. difficile* shedding (*P* = .04), whereas 14/81 (17%; 95% CI, 11%‐27%) mares and 19/82 (23%; 95% CI, 15%‐33%) foals that were negative at admission were subsequently positive (*P* = .44). One additional mare was positive on day 3 but an admission sample had not been collected, and the origin of the organism was not classified.

**FIGURE 1:**
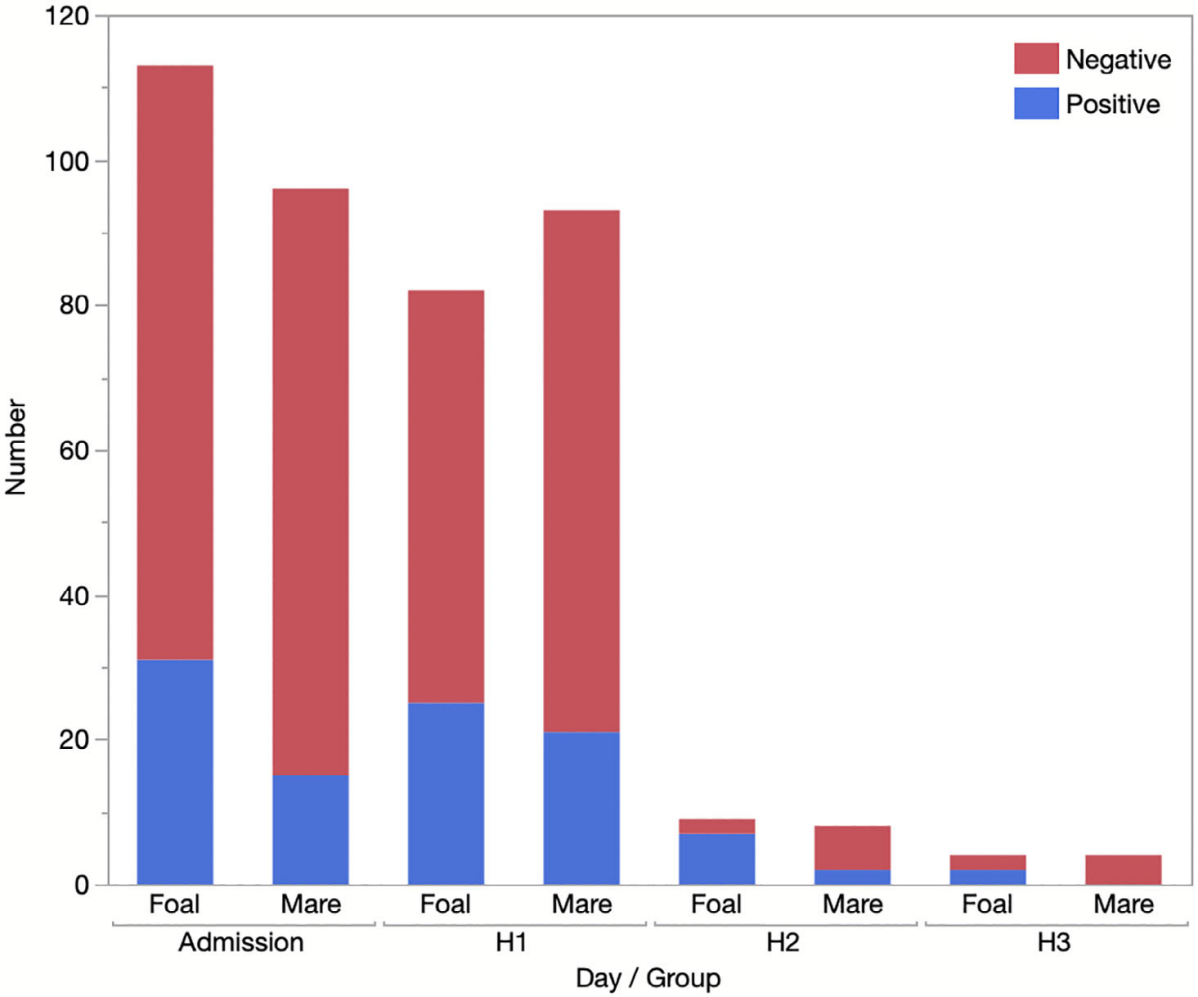
Isolation of *C. difficile* from mares (n = 97) and foals (n = 113) at admission and during hospitalization at an equine referral hospital. H1 = days 2‐4, H2 = days 5‐7, H3 = days 8‐10

There were 209 admission samples, 175 from H1, 17 from H2, and 8 from H3. Two foals were sampled twice during H2 (on both days 3 and 4). In both cases, results from the days were the same (negative). The day 4 samples were removed from statistical analyses so that only 1 sample was present for that sampling timepoint, but both were retained for descriptive analyses. Similarly, 1 mare was sampled on both days 2 and 3. Both were positive, and the day 2 sample was removed from statistical analysis. Results by sampling period are presented in Figure 1. No association was found between sampling day (admission, H1, H2, H3) and isolation of *C. difficile* overall (mares and foals combined, *P* = .13), or specifically for mares (*P* = .26) or foals (*P* = .26). When individual sampling points were compared to admission, no differences were found for mares (*P =* .3‐1.0). A significant difference from baseline was found in foals at H2 (*P* = .004, odds ratio [OR], 9.3; 95% CI, 1.8‐47) but not H1 (*P* = .75) or H3 (*P* = .57).

Fifteen foals had 2 positive samples during hospitalization. Eight were positive at both admission and H1. Four were positive at admission and H2, with 3 of those being negative and H1 and 1 having no H1 sample. Three were negative at admission and positive at H1 and H2. In only 6/15 (40%) foals was the same strain identified both times (5 ribotype 078 and 1 ribotype 012).

Two positive samples were identified from 8 mares. Five were positive at admission and H1, 2 were negative at admission but positive at H1 and H2, and 1 was positive on 2 different H1 samples (days 2 and 3). Four (50%) mares had ribotype 078 both times, with the other 4 having different ribotypes.

No associations were found between ribotype 078 (vs other strains combined) and location of suspected origin (community vs hospital) (*P* = .36), sampling day (*P* = .08) or group (mare vs foal; *P* = .17).

Longitudinal results for mares and foals that ≥2 samples collected are presented in Table 2. Twenty mares were negative at admission alongside positive foals and had subsequent rectal swabs collected. Four (20%) of those mares subsequently were positive, but only 1 of them shed the same strain (078) as the foal was shedding at admission.

**TABLE 2:** Subsequent culture results from mares and foals that had more than 1 sample collected during hospitalization and which had a negative admission culture

Admission status (n)	Outcome n (%)
Both negative (n = 39)	Both negative, 22 (56%) Only foal positive, 10 (27%) Only mare positive, 5 (13%) Both positive, 4 (10%)
Positive foal, negative mare (n = 20)	Mare negative, 16 (80%) Mare positive, 4 (20%)
Positive mare, negative foal (n = 7)	Foal negative, 5 (71%) Foal positive, 2 (29%)
Unaccompanied foal negative (n = 8)	Foal positive, 2 (25%)

Conversely, 7 foals that were negative at admission accompanied positive mares. Two (29%) of these foals subsequently were positive, but in both instances, the foal shed a different strain than the mare harbored at admission. In 1 pair, both the mare and foal harbored the same strain on day 3, but it was different from the strain the mare shed at admission.

**FIGURE 2:**
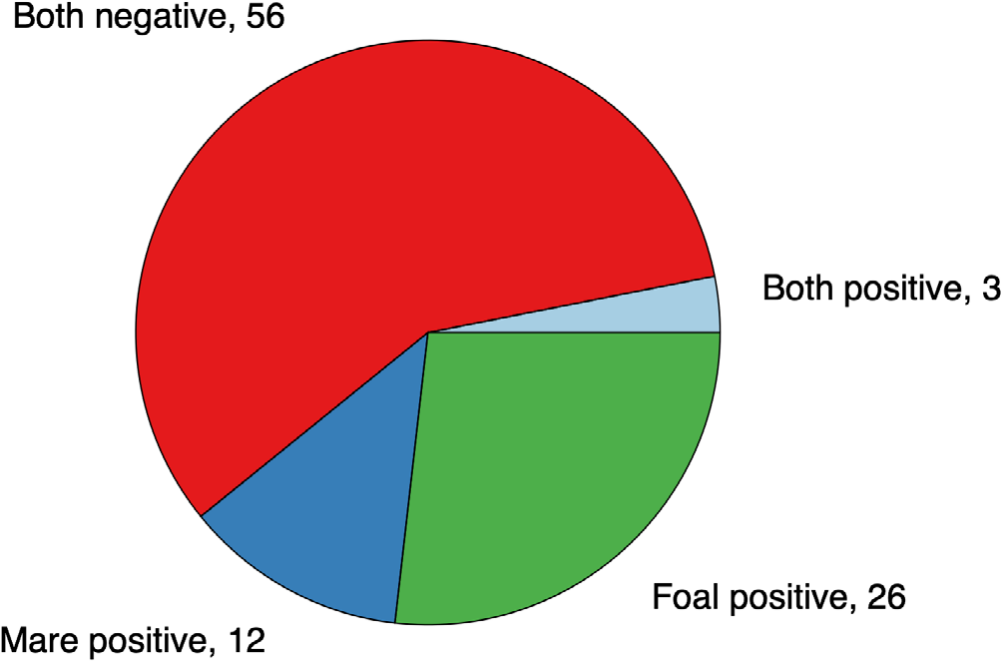
*C. difficile* shedding status of 97 mare‐foal pairs at admission to an equine referral hospital

Three mare and foal pairs both were shedding *C. difficile* at admission (Figure 2). In only 1 of those was the same strain (ribotype 078) identified in both the mare and foal.

**FIGURE 3:**
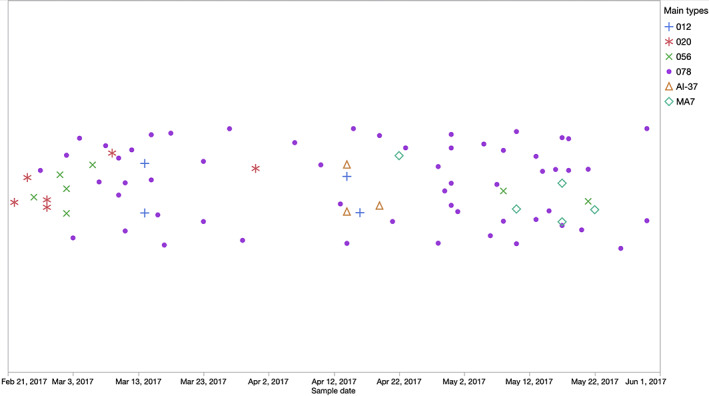
Timing of isolation of *C. difficile* ribotypes that were detected at least 3 times in mares and foals at an equine hospital

Some temporal clustering of ribotypes was apparent (Figure 3). For example, ribotype MA7 was found 5 times, with an admission positive sample on April 22 followed by 4 HA positives on May 10, May 17 (mare and foal pair), and May 22. Similarly, ribotype 020 was identified 6 times, with CA positive samples February 22 and 24, followed by HA positives on February 27 (mare and foal), March 9, and March 31.

## 4 DISCUSSION

The rate of *C. difficile* shedding at the time of admission was high in our study; but the study population must be considered. All mares were in the immediate postpartum period and alterations in the gut microbiota in the peripartum period have been reported in mares,^19^ which likely would predispose to transient shedding of *C. difficile*. Furthermore, higher rates of *C. difficile* shedding also have been reported in neonates of various species.^20^, ^21^, ^22^, ^23^ Thus, higher rates of shedding in our population compared to studies of other equine populations may not be surprising because the peri‐partum stressors and physical changes in mares, and the developing gastrointestinal microbiota in neonates, likely facilitate colonization with enteric opportunists such as *C. difficile*. The prevalence of shedding by foals also was similar to or higher in our study compared to what has been observed in other studies of foals.^1^, ^11^, ^13^ The young age of our foals and their compromised health status are other plausible predisposing factors. Although limited clinical data are available, all foals were critically ill with suspected noninfectious disease, based on their admission to the intensive care unit (rather than the general ward or isolation unit). Many could have received antimicrobials before referral and most would have been treated with antimicrobials during hospitalization.

Based on close contact between mares and foals, and the normal coprophagic behavior of foals, it is logical to assume there would be a strong influence on *C. difficile* transmission risk (especially mare to foal). One small earlier study using a less discriminatory typing method reported the same *C. difficile* strain in 36% of mare/foal pairs.^24^ This finding is in contrast to our study where only 3% of pairs were shedding at admission and in only 1 pair was the same strain shed by both mare and foal. Apparent transmission from mare to foal, or vice versa, occurred during hospitalization but was uncommon. Although numerically higher, no significant difference was found in *C. difficile* acquisition of foals from infected mares vs mares from infected foals. Thus, the exposure risk of mares and foals does not seem to be strongly linked, and other endogenous or exogenous (eg, environmental contamination, human cross‐contamination) factors may play important roles in the epidemiology of *C. difficile* in this rather unique subset of the population. Other potential factors such as severity of illness (and corresponding chance of coprophagia of the mare's feces or extent of contact with veterinary personnel) could not be analyzed.

Shedding was characterized as HA or CA using common definitions from human medicine.^18^ Although we can be confident that positive results at admission from horses that were not recently hospitalized indicate community acquisition, we have less confidence in subsequent samples because of the potential for false‐negative baseline cultures and lack of information about the incubation period or, more specifically, the time from oral exposure to shedding in adult horses and in foals. In an experimental study of foals, the meantime to first isolation of *C. difficile* was 48 hours,^25^ and thus false‐negative cultures are likely in a population of neonates that were exposed shortly after birth, before hospitalization. Therefore, although a standard approach to classification of HA and CA was used, this approach may overestimate HA rates and is a reason we have defined the term HA as “hospital‐associated” rather than “hospital‐acquired”. Clustering of some strains strongly suggested transmission within the hospital. However, because ribotype 078 dominated, clustering only could be detected with strains other than 078. More discriminatory approaches such as whole‐genome sequencing would be needed to better assess acquisition within the hospital setting. The identified clusters all were relatively small and self‐limiting (or controlled by routine infection control practices).

Changes in *C. difficile* shedding during hospitalization occurred. Although the cumulative prevalence increased throughout hospitalization, there was limited apparent difference in point prevalence over time and acquisition or loss of *C. difficile* shedding did not appear to be associated with duration of hospitalization, apart from a significantly higher prevalence in foals at H2 vs admission. Whether this finding represents a true increased risk during the day 4‐6 time period compared to other time periods requires further study. The sample sizes for post‐admission could have impacted our ability to detect changes at other time points.

The term “shedding” has been used in our study vs “colonization.” Although the term colonization is used commonly, it represents a state in which the bacterium is present and actively growing in the host. However, that conclusion is difficult to make, versus transient passage of the bacterium or metabolically inert bacterial spores. Whereas detection of *C. difficile* in feces likely reflects at least transient colonization, shedding has been used as a more accurate description of what can be assessed.

Limited longitudinal study of horses has been performed to characterize shedding dynamics. However, a study of 25 healthy horses that involved monthly fecal sample collection reported a cumulative horse‐level prevalence of 40% over the course of the year.^26^ However, the sample level prevalence was only 5.5%, and only once was the same strain found in consecutive monthly samples in the same horse. Another study reported significantly higher recovery of *C. difficile* by collection of 3 consecutive samples, with only 1/98 horses being positive on >1 sample.^26^ This finding is similar to our results, where shedding of individual strains was apparently limited, with transient shedding and in some instances apparent rapid acquisition of new strains. These findings suggest that *C. difficile* shedding is variable, sporadic, and dynamic in horses, rather than a situation in which a strain colonizes the gastrointestinal tract and persists. As is common, only 1 *C. difficile* isolate was typed from each positive sample. Although it often is assumed that multi‐strain colonization is rare, the potential for concurrent colonization with multiple strains must be considered. Therefore, it is impossible to definitively state that identification of new strains during hospitalization represents true HA acquisition vs identification of a strain that was present but overlooked initially as part of a coinfection. Fecal samples or rectal swabs also may under‐represent the true intestinal colonization rate. Sampling throughout the intestinal tract has yielded higher rates, but obviously is not possible for routine surveillance.^27^

Ribotype 078 predominated, but a wide range of ribotypes was identified. Ribotype 078 previously has been reported as the most common ribotype from horses in some,^26^, ^28^ but not all^7^, ^29^ studies. This toxinotype V, clade 5 strain^30^ is found commonly in food‐producing animals,^21^, ^28^, ^29^ but is relatively uncommon in humans and other domestic animals such as dogs. Reasons for species associations are unclear, as are the zoonotic disease risks posed by animals. Although ribotype 078 is an uncommon strain in humans, it can cause disease and is most commonly associated with CA CDI.^31^, ^32^, ^33^ Furthermore, 15/23 (65%) ribotypes found in our study, accounting for 90/103 (87%) isolates, were strains found in humans. This finding raises concern about the potential for zoonotic transmission, as well as human‐to‐animal transmission, but our study was not designed to address these issues. The second most common strain, ribotype 056, has been associated with disease in both people and animals, and, like ribotype 078, has been associated with CA CDI in people.^28^, ^29^, ^30^, ^34^, ^35^

There are various potential sources of *C. difficile* in veterinary hospitals, including horses, the environment, equipment, and personnel. Our study included only neonatal foals and their dams, and indirect transmission to or from horses in other parts of the hospital could have occurred. Testing of horses in other wards was not performed, and the relationship and cross‐transmission risk among different hospital wards are unknown.

Our study had some limitations. The study involved only a single veterinary hospital in a single region, and transmission dynamics may be heterogenous geographically. Our study population was almost exclusively Thoroughbred horses, and breed differences could exist. Furthermore, the study population was mares and foals from breeding farms, and *C. difficile* transmission may differ on other types of farms. Our study provides useful information about the dynamics of *C. difficile,* but whether results would be similar in different populations requires further investigation.

*Clostridium difficile* is an important pathogen in adult horses and foals, and our study emphasizes the complexity surrounding the epidemiology of this opportunistic pathogen. It can be commonly found, transiently present and cluster within a facility in the absence of identifiable disease occurrences or clusters. These features highlight the importance of routine infection control and antimicrobial stewardship to try and limit transmission of this pathogen. Our results contribute to a better understanding of the epidemiology of *C. difficile* in hospitals and interpretation of routine or outbreak surveillance.

## CONFLICT OF INTEREST DECLARATION

Authors declare no conflict of interest.

## OFF‐LABEL ANTIMICROBIAL DECLARATION

Authors declare no off‐label use of antimicrobials.

## INSTITUTIONAL ANIMAL CARE AND USE COMMITTEE (IACUC) OR OTHER APPROVAL DECLARATION

Approved by University of Guelph Animal Care Committee.

## HUMAN ETHICS APPROVAL DECLARATION

Authors declare human ethics approval was not needed for this study.
